# The Polish Version of the Alberta Infant Motor Scale: Cultural Adaptation and Validation

**DOI:** 10.3389/fneur.2022.949720

**Published:** 2022-07-28

**Authors:** Małgorzata Eliks, Anna Sowińska, Ewa Gajewska

**Affiliations:** ^1^Chair and Clinic of the Developmental Neurology, Poznan University of Medical Sciences, Poznań, Poland; ^2^Doctoral School of Poznań University of Medical Sciences, Poznań, Poland; ^3^Department of Computer Science and Statistics, Poznan University of Medical Sciences, Poznań, Poland

**Keywords:** Alberta Infant Motor Scale, infant, motor development, reliability, validation

## Abstract

The Alberta Infant Motor Scale (AIMS) is a diagnostic tool for the assessment of the motor performance of infants from the time of birth, to the period of independent walking (0–18 months). This study aims to derive a Polish version of the AIMS through its cultural adaptation and validation. The study included 145 infants aged 0–18 months, who were divided into four further age groups: 0–3 months, 4–7 months, 8–11 months, and older than 12 months. The validation was based on an analysis of intrarater and interrater reliability values, as well as concurrent validity, using the gross motor scale of Peabody Developmental Motor Scales-2 (PDMS-2). The total Intraclass Correlation Coefficient (ICC) for intrarater reliability was 0.99 (ICC range in positions was 0.87–0.99, in subgroups was 0.91–0.99), while in particular positions, the ICC ranges were as follows: prone 0.97–0.99, supine 0.94–0.99, sitting 0.95–0.99, and standing: 0.63–0.99. The total ICC for interrater reliability was 0.99 (ICC range in positions was 0.98–0.99, in subgroups was 0.91–0.99), while in particular positions, the ICC ranges were as follows: prone 0.95–0.99, supine 0.93–0.96, sitting 0.93–0.98, standing 0.91–0.98. Only the standing position was analyzed for the subgroup of participants over 12 months old. The Spearman correlation between the Polish version of the AIMS and the gross motor scale of PDMS-2 was significant in the total population (r = 0.97, *p* < 0.0001) and in subgroups (r = 0.79–0.85, *p* < 0.0001). The results of our study confirm that the Polish version of the AIMS is reliable for infants aged 0–18 months and can be applied to this population for clinical and scientific purposes.

**Trial Registry:**

ClinicalTrials.gov ID NCT05264064, URL https://clinicaltrials.gov/ ct2/show/NCT05264064.

## Introduction

During infancy, motor performance is a reliable manifestation of the functionality and integration of the central nervous system (1–5). Hence, the monitoring of infant motor development allows for detecting delays and disturbances and enabling, if necessary, early therapeutic interventions to prevent further structural and functional disorders (6). However, the developmental assessment should be performed by using standardized tools, which need to undergo cultural adaptation and validation. This process is necessary to ensure their reliability in countries, cultures, populations, and languages different than initially intended (7, 8). So far, there has been no validated and standardized Polish version of any worldwide used scale for infant motor development.

The Alberta Infant Motor Scale (AIMS) is a diagnostic tool for the developmental evaluation of infants from the time of birth, to the period of independent walking (0–18 months), based on the observation of spontaneous motor performance (9, 10). It is based on several assumptions of the neuromaturational model and the concepts of the dynamic system development theory (9, 10). The AIMS was created in the early 1990s by Piper and Darrah, and validated and standardized on the Canadian population (9, 10). Moreover, a 2014 re-evaluation of the scale noted that the normative values in this population remained stable (11). The intended uses of the AIMS comprise 1) identification of infants with motor delay, 2) providing medical professionals and parents information on motor achievements of the infant (both currently developing activities and those not observed in the infant's repertoire), 3) measurement of motor performance over time or before and after an intervention, and 4) as a research tool for the estimation of rehabilitation program efficacy in infants with motor delays (9, 10). The scale has been used (as an outcome measure) in numerous studies on healthy infants (12–14) and those affected by or at risk of developmental disorders. These included preterm birth (15), perinatal hypoxic-ischemic encephalopathy (16, 17), cystic periventricular leukomalacia (18), cardiac surgery (19), univentricular heart (20), positional plagiocephaly (21, 22), torticollis (23), positional asymmetry (24), Down syndrome (25), and infected with the Zika virus (26). So far, research on the reliability and validity of the AIMS has been performed in Japanese (27), Chinese (28), Brazilian (29), Spanish (30), Thai (31–33), Greek (34), Dutch (35), Flemish (14), and Serbian (36) sample groups. However, there has not yet been a study conducted on any Middle European population. According to the studies mentioned above, the psychometric properties of the AIMS are commendable. Values of the intrarater reliability and interrater reliability were established above 0.9, while the concurrent validity with other neurodevelopmental scales, such as the Bayley Scales of Infant and Toddler Development III (BSID-3) and the Peabody Developmental Motor Scales II (PDMS-2), were 0.95–0.98 and 0.90–0.99, respectively. Besides its applicability and psychometric values, the construction of the AIMS makes it approachable for both novice and experienced researchers (37). According to Snyder et al., the ICC between raters of varying experience was estimated at 0.98 in the assessment of infants younger than 10 months (37). However, in the evaluation of an older infant group, significant differences between raters were found, possibly due to varied motor performance at this age, requiring more rater experience for proper assessment (37). Moreover, the authors of the AIMS recommend researchers to obtain previous experience in infant developmental assessment, before the use of AIMS for clinical and research purposes (10).

This study aims to derive a Polish version of the AIMS through its cultural adaptation and validation. This process was based on an analysis of the intrarater and interrater reliability values, as well as concurrent validity, using PDMS-2.

## Materials and Methods

### Participants

The study included 145 infants aged 0–18 months who were divided into four age groups: 0–3 months, 4–7 months, 8–11 months, and older than 12 months. The participants were recruited *via* targeted advertisements on parenting-related websites, antenatal classes, nurseries, and neonatal and pediatric outpatient departments in the Greater Poland region. The study was conducted between November 2020 and September 2021. The inclusion criteria were 1) a gestational age between 37 and 42 weeks and 2) birth weight of ≥2500 g, 3) 5-min Apgar score ≥8. In turn, the exclusion criteria comprised 1) a gestational age <37 weeks, a birth weight <2500 g, 5-min Apgar score <8, and 4) the presence of any neurological, orthopedic, genetic, metabolic, and sensory disorders. All parents or caregivers expressed their written consent to their children's participation in the study. The research was conducted in agreement with the Declaration of Helsinki and the Poznan University of Medical Sciences Bioethics Committee (approval no. 1034/19).

### Study Design

The translation, cultural adaptation, and validation were conducted according to existing recommendations (8, 38). Firstly, two forward translations of the original AIMS were performed by two translators, one of them had a medical background (8, 38). The two versions were compared and consolidated (8, 38). Then, a double back translation was performed by different translators (also containing one individual with medical knowledge). Furthermore, a synthesis of the two back translations and a comparison with the original version of the AIMS were performed by the research committee of the clinic (8, 38). This process allowed us to confirm the semantic, idiomatic, experiential, and conceptual equivalence of the Polish version (8). A pilot testing of the final version was carried out on a group of 40 infants. Then, psychometric properties of the final version of the Polish AIMS scoresheet, such as intrarater and interrater reliability values and concurrent validity, were examined. The study was conducted by two pediatric physiotherapists with a minimum of 7 years of experience in the developmental assessment and therapy of infants. The examination methodology was concordant with the recommendation of the authors of the AIMS and the PDMS-2 (10, 39). A fully fed and well-rested infant wearing a diaper was placed on a rehabilitation table or mat in a warm room during the assessment. The intrarater reliability test included two assessments (with 1-month intervals) performed by one researcher—a pediatric physiotherapist (Rater A). For the second assessment, the videos of spontaneous motor performance of infants were recorded during the examination. The repeated tests of the same rater were performed within a 1-month interval to avoid a risk of assessment bias (28, 30, 32). The interrater reliability involved assessments by two researchers—pediatric physiotherapists (Rater A and Rater B). Furthermore, the Gross Motor Scales of the PDMS-2 was administered to estimate concurrent validity (participants at the age of 0–12 months).

### Instruments

#### Alberta Infant Motor Scale

The AIMS scoresheet consists of 58 items at four positions (21 in prone, 9 in supine, 12 in sitting, and 16 in standing) (9). The components assessed for each item are based on three elements of movement: weight-bearing, posture, and antigravity movements (9). A drawing of the infant's position accompanies every item (9). The evaluation of the infant is based on the observation of spontaneous movement with minimal handling, e.g., encouragement using a toy (9). The examiner had to identify the least and the most mature items in every position—these constituted the developmental “window” and then to score every item in the “window” as “observed” or “not observed” (9). Each item below the least mature was treated as “observed.” The scoring was dichotomous for each item—“observed” (1 point) or “not observed” (0 points) (9). The sum of all the items in every position yielded the total raw score, which may be converted into percentile ranks (with 1-month age group intervals) (10). The assessment lasts 20–30 min and may be performed based on direct observation or video recording.

#### Peabody Developmental Motor Scales-2

Three subtests of the Gross Motor Scale of the PDMS-2 were used: reflexes (administered in infants under 12 months)–8 items, stationary–30 items, and locomotion–89 items (39). Each item was rated on a three-point scale: 0—the child could not or would not attempt the item or the attempt did not indicate that the skill was emerging, 1—the child's performance indicated a clear resemblance to the item mastery criteria but did not fully meet the criteria and 2—the child performed the item according to the criteria specified for mastery (39). The raw scores of the subtests on the PDMS-2 can be summed to yield the Gross Motor Quotient (39). The examination lasted approximately 20–30 min (39).

### Data Analysis

Statistical analysis was carried out using the Statistica software (data analysis software system; TIBCO Software Inc., 2017; v.13). Categorical variables were described as proportions. Means and standard deviations (SDs) were calculated for the continuous data with normal distribution, estimated using Shapiro-Wilk's test. Non-normally distributed data were presented as medians and minimum-maximum ranges. Intrarater and interrater reliability values for measuring the variability introduced by different observations and observers were examined *via* calculating the Intraclass Correlation Coefficient (ICC) and the 95% confidence interval (95% CI) of the ICC for the positions, as well as total scores for the four studied age groups. It has been determined that values of ICC <0.5 indicate reliability as poor, 0.5–0.75 as moderate, 0.75–0.9 as good, and >0.90 as excellent (40).

Moreover, intrarater and interrater reliability values were also analyzed using Bland-Altman plots. Correlations between Gross Motor Scales of PDMS-2 and the AIMS were evaluated using the Spearman rank coefficient. Additionally, the differences between the assessments of the rating (Rater A) and raters (Rater A and Rater B) were evaluated using the Wilcoxon test.

## Results

**Figure 1:**
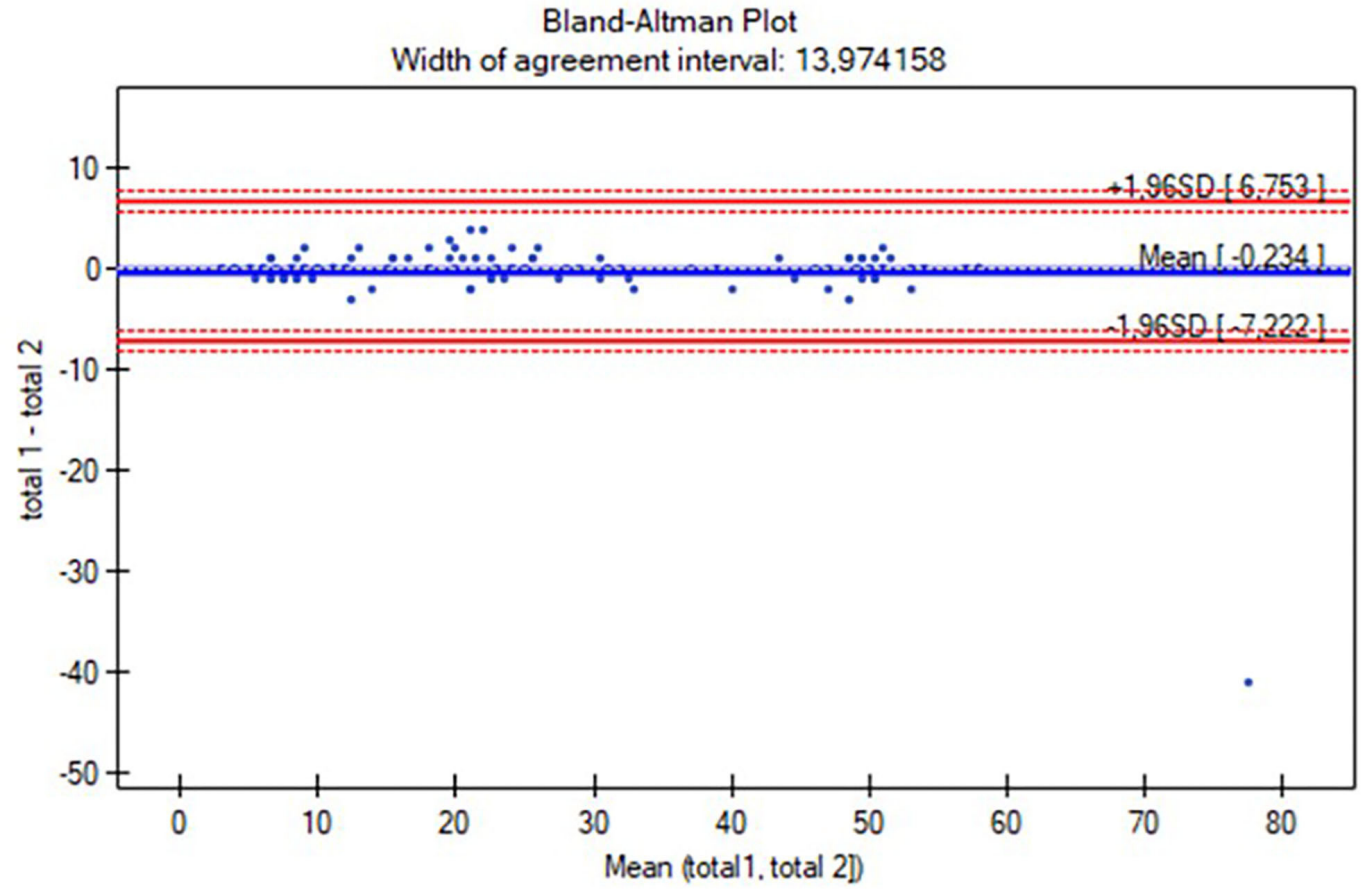
The Bland-Altman plot of the total Alberta Infant Motor Scale (AIMS) score intrarater reliability. Mean (total 1, total 2): average of measurements for compared methods; total 1—total 2: the difference between measurements for compared methods; mean difference ± 1.96 SD: 95% limits of agreement.

**Figure 2:**
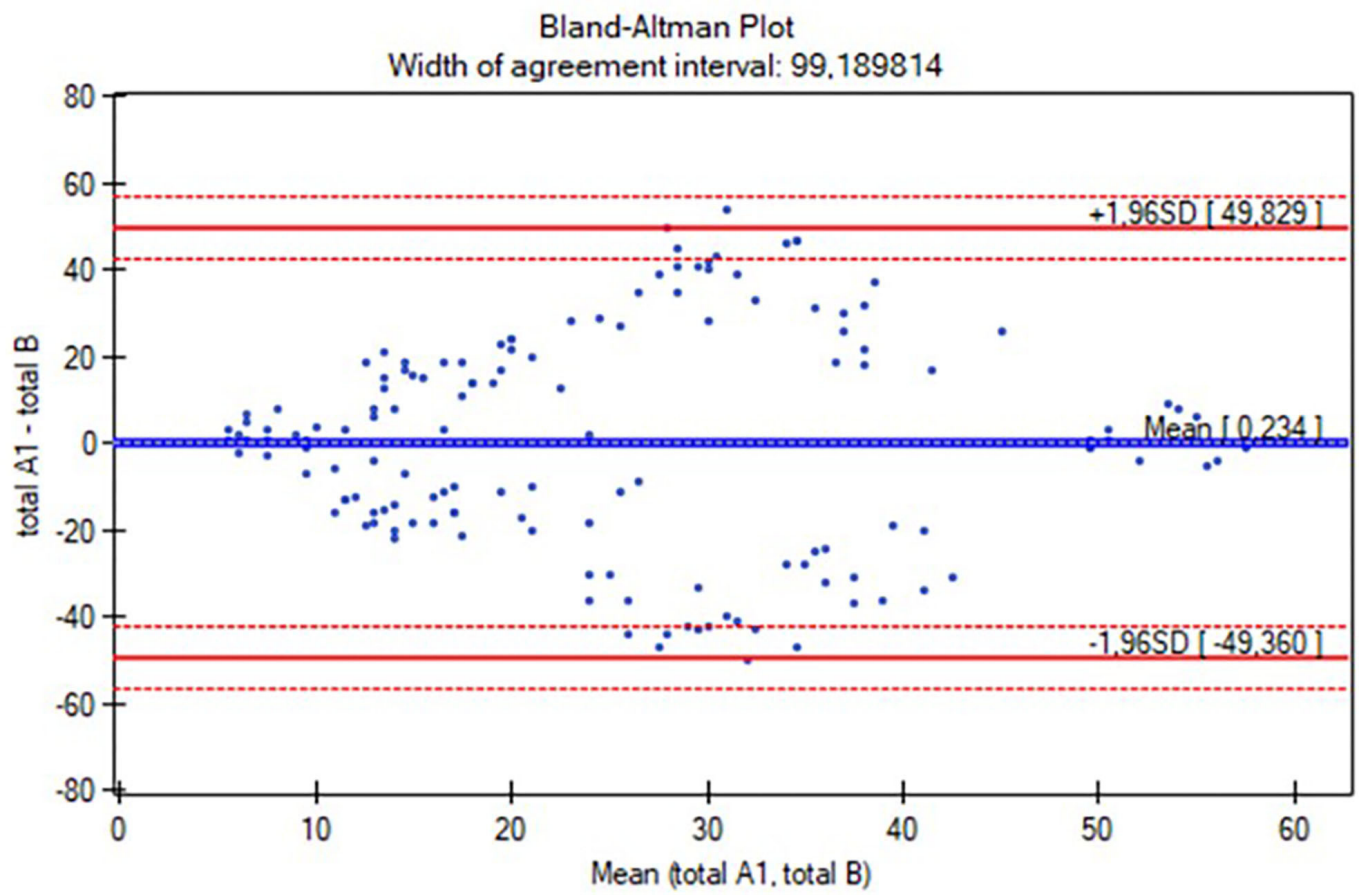
The Bland-Altman plot of the total Alberta Infant Motor Scale (AIMS) score interrater reliability. mean (total A1, total B): average of measurements for compared methods; total A1—total B: the difference between measurements for compared methods; mean difference ± 1.96 SD: 95% limits of agreement.

The Polish version of the AIMS scoresheet was developed based on the study proceedings. The characteristics of the participants are listed in Table 1. The reliability study included 145 participants (aged 0–18 months), while the concurrent validity study involved 127 individuals (aged 0 to <12 months). The study group was analyzed all together and with a division into four subgroups: 0–3 months (*n* = 56), 4–7 months (*n* = 40), 8–11 months (*n* =31), and over 12 months (*n* = 18; Table 1). Table 2 presents the results of intrarater and interrater reliability values. The total ICC in intrarater reliability was 0.99 (ICC range in positions was 0.87–0.99 and in subgroups was 0.91–0.99), while in particular positions the ICC ranges were as follows: prone 0.97–0.99, supine 0.94–0.99, sitting 0.95–0.99, and standing 0.63–0.99 (Table 2). The total ICC was 0.99 (ICC range in positions was 0.98–0.99 and in subgroups 0.91–0.99), while in particular positions the ICC ranges were as follows: prone 0.95–0.99, supine 0.93–0.96, sitting 0.93–0.98, and standing 0.91–0.98. Only the standing position was analyzed for the subgroup of participants over 12 months (all children achieved maximal scores while prone, supine, and sitting; Table 2). No significant differences in total score and subgroup results (except for the supine position in the 4–7 months subgroup, *p* = 0.02) were found between the assessments (Wilcoxon test, *p* > 0.05), both when examined by the same and two different raters. The consistency between the intra-/interrater assessments was significant in all variables (*p* < 0.05). Furthermore, intrarater and interrater reliability values of the total AIMS scores were also examined for all participants using the Bland-Altman analysis (Figures 1, 2). For scorings by Rater A (Figure 1), the mean difference was 0.23, while 95% limits of agreement were between −7.22 and 6.75. For Rater A and Rater B (Figure 2), the mean difference was 0.23, with 95% limits of agreement between −49.36 and 49.83. The Spearman correlation between the Polish version of the AIMS and the gross motor scale of PDMS-2 was significant in total population (r = 0.97, *p* < 0.0001), as well as subgroups: 0–3 months (r = 0.79, *p* < 0.0001), 4–7 months (r = 0.85, *p* < 0.0001), and 8–11 months (r = 0.83, *p* < 0.0001).

**Table 1:** Participant characteristics.

		***n* (%)**	**mean (SD)**	**Median**	**min-max**
Sex	female	68 (46.9)			
	male	77 (53.1)			
Age				182 days	14 days−18 months 14 days
	0–3 mo	56 (38.6)			
	4–7 mo	40(27.6)			
	8–11 mo	31 (21.4)			
	>12 mo	18 (12.4)			
Birth weight (g)			3453.3 (415.9)	3455	2500.0–4690.0
5-min Apgar score	9	7 (4.8)			
	10	138 (95.2)			
Gestational age (wk)	37– <38	8 (5.5)			
	38– <39	20 (13.9)			
	39- <40	47 (32.4)			
	40– <41	26 (17.9)			
	41– <42	35 (24.1)			
	42	9 (6.2)			
Birth method	natural	98 (67.6)			
	cesarean section	47 (32.4)			
Birth order	1	96 (66.2)			
	2	39 (26.9)			
	3	8 (5.5)			
	4	-			
	5	2 (1.4)			
Age of mother at birth (y)			31.5 (3.9)	31	22–44
Age of father at birth (y)			33.6 (4.6)	33	25–57

*mo, months; wk, weeks; y, years; SD, standard deviation*.

**Table 2:** The results of intrarater and inter-rater reliability.

	**Intrarater reliability**				**Interrater reliability**			
	**ICC**	**95% CI**	***p***	***p* (Wilcoxon test)**	**ICC**	**95% CI**	***p***	**p (Wilcoxon test)**
**Total group**								
prone	0.99	0.99–0.99	<0.000001	0.77	0.99	0.99–0.99	<0.000001	0.77
supine	0.99	0.99–0.99	<0.000001	0.24	0.99	0.99–0.99	<0.000001	0.24
sitting	0.99	0.99–0.99	<0.000001	0.61	0.99	0.99–0.99	<0.000001	0.61
standing	0.87	0.82–0.91	<0.000001	0.94	0.99	0.98–0.99	<0.000001	0.94
total	0.99	0.99–0.99	<0.000001	0.88	0.99	0.99–0.99	<0.000001	0.88
**0–3 months**								
prone	0.97	0.95–0.98	<0.000001	0.77	0.96	0.93–0.97	<0.000001	0.77
supine	0.98	0.97–0.99	<0.000001	0.59	0.96	0.93–0.98	<0.000001	0.59
sitting	0.96	0.92–0.97	<0.000001	0.07	0.93	0.88–0.96	<0.000001	0.06
standing	0.85	0.75–0.91	<0.000001	0.74	0.92	0.86–0.95	<0.000001	0.74
total score	0.99	0.98–0.99	<0.000001	0.34	0.98	0.97–0.99	<0.000001	0.34
**4–7 months**								
prone	0.99	0.98–0.99	<0.000001	0.35	0.98	0.96–0.99	<0.000001	0.35
supine	0.94	0.89–0.97	<0.000001	0.02	0.93	0.86–0.96	<0.000001	0.02
sitting	0.99	0.97–0.99	<0.000001	0.59	0.99	0.98–0.99	<0.000001	0.59
standing	0.97	0.94–0.98	<0.000001	0.18	0.92	0.85–0.96	<0.000001	0.18
total score	0.99	0.98–0.99	<0.000001	0.50	0.99	0.98–0.99	<0.000001	0.05
**8–11 months**								
prone	0.99	0.99–0.99	<0.000001	0.14	0.99	0.99–0.99	0.000921	0.14
supine	0.99	0.98–0.99	<0.000001	too few values	No analysis due to low data variability			
sitting	0.99	0.98–0.99	<0.000001	0.35	0.99	0.97–0.99	<0.000001	0.35
standing	0.64	0.25–0.82	0.003461	0.48	0.98	0.97–0.99	0.001362	0.48
total score	0.92	0.83–0.96	<0.000001	0.10	0.99	0.99–0.99	<0.000001	0.10
**>12 months**								
standing score	0.99	0.98–0.99	<0.000001	0.68	0.91	0.77–0.96	0.00004	0.68

*ICC, Intraclass Correlation Coefficient; CI, Confidence Interval*.

## Discussion

We performed cultural adaptation and validation of the Polish version of the AIMS through an analysis of the intrarater and inter-rater reliability values, as well as concurrent validity, using the gross motor scale of the PDMS-2. Our study estimated the overall intrarater and inter-rater reliability ICC values of 0.99. This result is consistent with the original version of AIMS validated in the Canadian population (the intrarater and inter-rater reliability values were also set at 0.99) (9). No significant score differences between the same rater and two raters were found. A value of 0.97 in concurrent validity is concurrent with the studies by Piper and Darrah, Snyder et al., and Wang et al. (9, 28, 37). Moreover, we also analyzed a coincidence between scores of the same rater and between raters in age subgroups and subscales. We divided our cohort into age subgroups (0–3 and 4–7 months), with consideration of the methodology of previous studies (30, 32) and the expectation of motor milestone achievement (8–11 months and above 12 months), as indicated by norms devised by Piper and Darrah and the World Health Organization (WHO) (10, 41). In detailed analyses, in positions and subgroups, values mostly exceeded 0.9 (except for standing position intrarater reliability in the 8–11 months group). This result may be explained by the requirement of the rater's personal involvement, e.g., safeguarding an infant who starts to sit or stand on the examination mat, which could result in a difference between a real-time assessment and video scoring.

We examined 145 full-term infants from the age of 2 weeks to 18 months. Some previous studies only included full-term infants (29), others were based exclusively on infants at risk (28, 30, 31, 36), while one study comprised both full-term and preterm participants (28, 36). The sample size varied from 30 to 259 in reliability estimation and 30 to 86 in concurrent validity scoring. Most of the studies included participants at the age of 0–18 months, while the research by Lackovic et al. was based on infants up to 14 months old, and Wang et al. only included individuals aged 0–9 months (28, 36). Nonetheless, according to the authors of the AIMS and the WHO norm for independent walking, which assumes the achievement of this motor milestone at the 18th month of life, it seems reasonable to involve participants up to this age in validation studies (9, 40). The first validation of AIMS was performed in the Thai population of 45 premature infants (allocated into three age groups: 0–3 months, 4–7 months, and 8 months and older). The overall ICC values for intrarater reliability and inter-rater reliability were 0.97–0.99, while in subgroups and subscales, they varied from 0.73 to 0.99 (31). Uesugi et al. performed validation in Japan in a group of 40 healthy infants from the age of 22 days to 17 months (27). The results were analyzed as AIMS total scores and subscales in two age groups (0–7 months and 8 months and older). The intrarater reliability exceeded 0.94 in an assessment of six different raters (27). The authors did not analyze the supine position in infants above 8 months because ICC scores were almost the same (27). Similarly, in our study, we could not analyze the prone, supine, and sitting ICC scores in the group of 12 months or older infants due to the measurement coincidence. So far, the largest study was carried out by Valentini et al. on Brazilian infants (aged 0–18 months) (29). The authors involved 259 participants for intrarater and inter rater reliability values (based on three raters). The intrarater and inter rater reliability values were set at 0.98, 0.91–0.99 (in positions), and 0.86–0.99 (depending on the combination of raters and positions) (29). In turn, Morales-Montforte et al., in the validation of the Spanish version of AIMS, analyzed the ICC in the total group (50 individuals at the age of 0–18 months, all at developmental risk) and within age subgroups (0–3, 4–8, and 9–18 months). The intrarater reliability ranged from 0.94 to 1.00, while the consistency between the two raters was estimated at 0.95–1.00 (30). The validation in the Chinese population was also performed on infants at risk (28). The cohort of 50 individuals was additionally divided into two age subgroups: 0–3 and 4–9 months (28). The intrarater and interrater ICC values (based on three raters) were similarly high: 0.81–0.99 and 0.98–0.99, respectively (28). Aimsamrarn et al. carried out the second Thai validation, such as translation and cultural adaptation, on a group of 30 healthy infants (divided into six age groups: 1–3, 4–6, 7–9, 10–12, 13–15, and 16–18 months) (32). Similarly, the intrarater and interrater reliability values were relatively high (0.98–0.99) (32). However, the authors only reported results in the total group, without the division into subgroups and positions. The newest study on the Serbian AIMS version, in the group of 60 infants (at a developmental risk), analyzed in total and in three age subgroups (0–3, 4–7, and 8–14 months), indicated variability in intrarater (with an interval of 5 days) and interrater reliability values (two raters) depending on age and subscales (36). The ICC was more than 0.75 in all measurements, except for the standing position in the group of 4–7 months (the ICC = 0.655 in rating by the same rater and two raters) and for sitting and standing positions in the group aged 0–3 months (ICC = 0.671 and 0.725, respectively, between rating by the same rater) (36). In our study, the lowest ICC (0.64) was described in intrarater reliability in the standing position for the 8–11 months old group. Notably, while the methodology of studies validating the various AIMS versions varied to a greater or lesser extent, their psychometric values were relatively similar.

Similarly to the authors of the original AIMS, we performed a concurrent validity study in infants up to 12 months of age, using the gross motor scale of the PDMS, particularly its contemporary version—PDMS-2. Our correlation results were concurrent with the studies by Piper and Darrah, Snyder et al., and Wang et al. (9, 28, 37).

A notable strength of this study is the sample size, which included participants in the whole age range of 0–18 months. The process of cultural adaptation and validation was conducted with canonical standards. Thus, the Polish scoresheet of the AIMS can be applicable in further research. Despite meticulous preparation of the methodology of our study, we realize that there are some limitations to its design. Firstly, the study was conducted in a single center, located in one of the biggest cities in Poland, which limited the variety of participants (e.g., a lack of individuals from rural areas or other districts of the country). The second limitation was a relatively small number of infants older than 12 months included in the study, due to recruitment difficulties associated with this age group. We assume that it could have been caused by the fact that in Poland, the maternity leave is limited to 12 months, after this time, mothers usually return to work while the children begin to attend nurseries.

The results of our study confirm that the Polish version of the AIMS is reliable for infants aged 0–18 months and can be applied to this population for clinical and scientific purposes.

## Data Availability Statement

The raw data supporting the conclusions of this article are not publicly available due to privacy but are available from the corresponding author on reasonable request.

## Ethics Statement

The study was reviewed and approved by Poznan University of Medical Sciences Bioethics Committee (approval no. 1034/19). Written informed consent to participate in this study was provided by a participant's parent or caregiver.

## Author Contributions

ME: research concept and design, collection of data, interpretation of data, writing the article, and final approval of the article. AS: data analysis and interpretation of results, critical revision of the article, and final approval of the article. EG: research concept and design, collection of data, critical revision of the article, and final approval of the article. All authors contributed to the article and approved the submitted version.

## Funding

Research was financed from the large research grant from statutory funding for young researchers—doctoral students for 2021. Poznan University of Medical Sciences, grant number [SDUM-GB9/03/21].

## Conflict of Interest

The authors declare that the research was conducted in the absence of any commercial or financial relationships that could be construed as a potential conflict of interest.

## Publisher's Note

All claims expressed in this article are solely those of the authors and do not necessarily represent those of their affiliated organizations, or those of the publisher, the editors and the reviewers. Any product that may be evaluated in this article, or claim that may be made by its manufacturer, is not guaranteed or endorsed by the publisher.
