# Erratum: The polish version of the Alberta Infant Motor Scale: Cultural adaptation and validation

**DOI:** 10.3389/fneur.2023.1136965

**Published:** 2023-01-18

**Authors:** Frontiers Production Office

**Affiliations:** Frontiers Media SA, Lausanne, Switzerland

**Keywords:** Alberta Infant Motor Scale, infant, motor development, reliability, validation

Due to a production error, the wrong ethics statement was used. The correct statement is found below:

## Ethics statement

The study was reviewed and approved by Poznan University of Medical Sciences Bioethics Committee (approval no. 1034/19). Written informed consent to participate in this study was provided by a participant's parent or caregiver.

The publisher apologizes for this error and states that this does not change the scientific conclusions of the article in any way. The original article has been updated.

